# Supplementary figures and images for: Enamel histomorphometry, growth patterns and developmental trajectories of the first deciduous molar in an Italian early medieval skeletal series
Source: PLoS One. 2024 Dec 5;19(12):e0304051. doi: 10.1371/journal.pone.0304051 (PMC11620606; doi:10.1371/journal.pone.0304051)

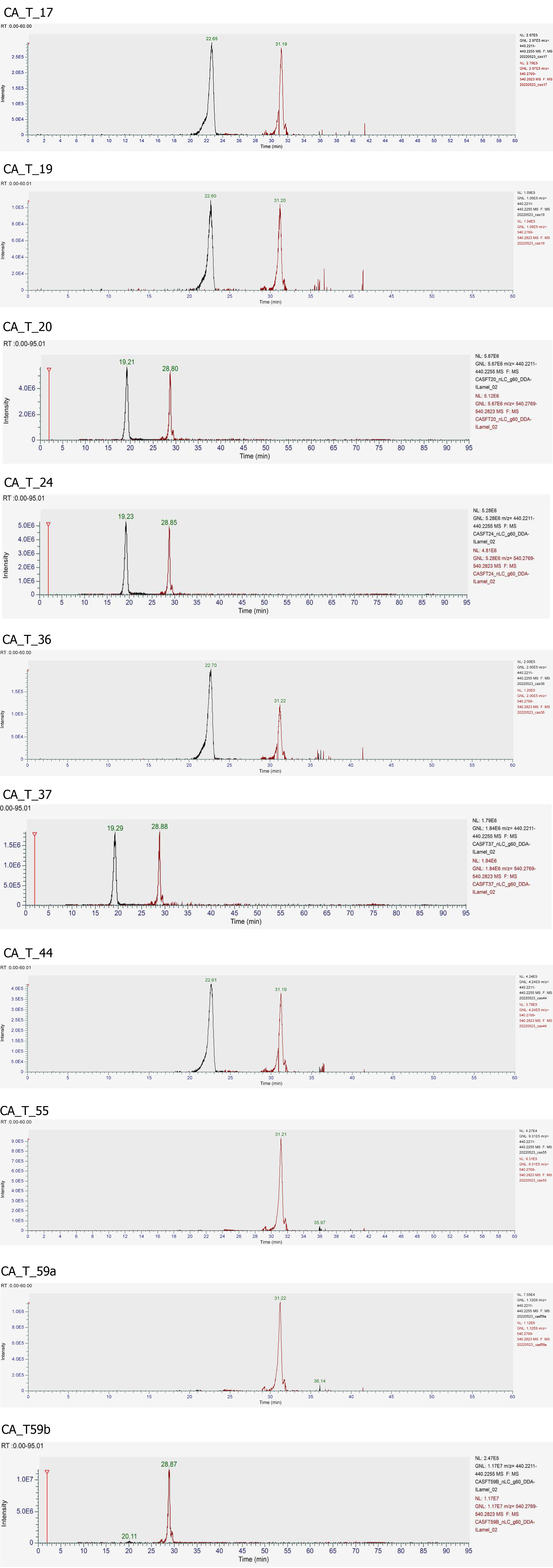

Supplement: S1 Fig — The presence of both isoforms, AMELX (time ~30 min) and AMELY (time ~20 min), determines sex as male. Conversely, the presence of the only AMELX isoform assesses the sex as female. From Casalmoro 3 individuals are estimated as male and 5 as female. (TIF) [file pone.0304051.s001.tif]

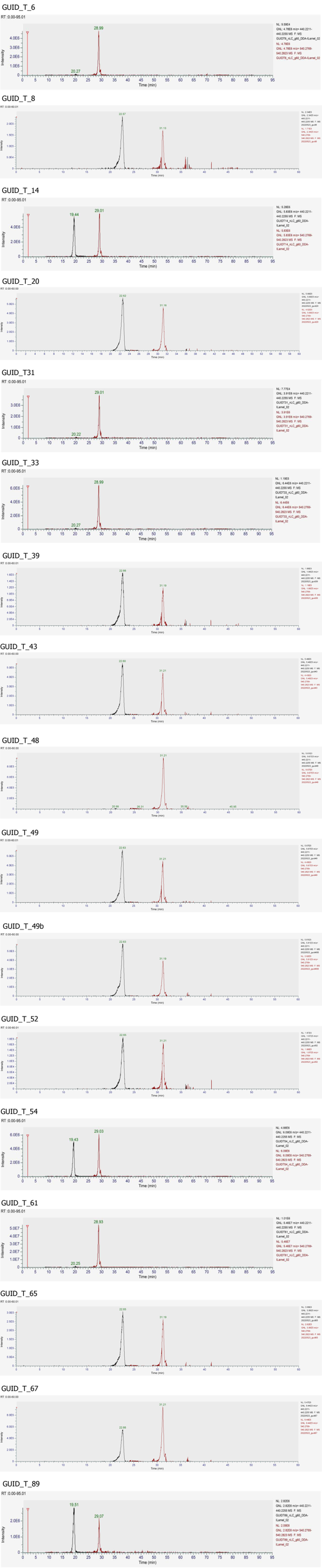

Supplement: S2 Fig — The presence of both isoforms, AMELX (time ~30 min) and AMELY (time ~20 min), determines sex as male. Conversely, the presence of the only AMELX isoform assesses the sex as female. From Guidizzolo were identified 5 females and 12 males. (TIF) [file pone.0304051.s002.tif]

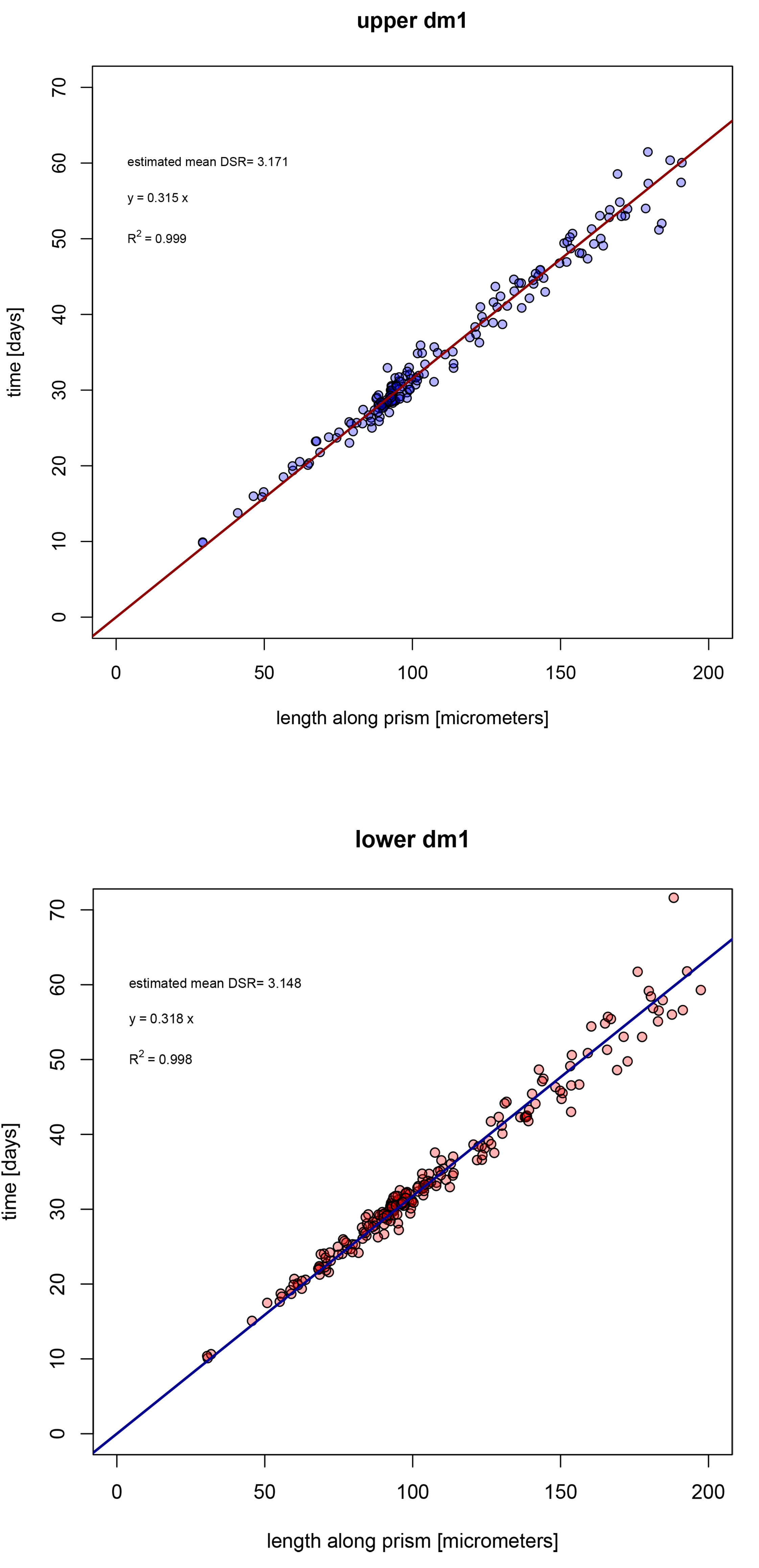

Supplement: S3 Fig — The regression formula for upper molars is Y = 0.315 X and the estimated mean DSR was 3.17 μmday-1. The regression formula for lower molars is Y = 0.318 X and the estimated mean DSR was 3.15 μmday-1. (TIF) [file pone.0304051.s003.tif]

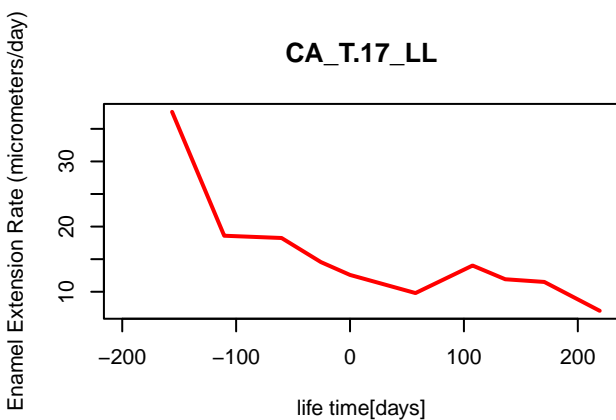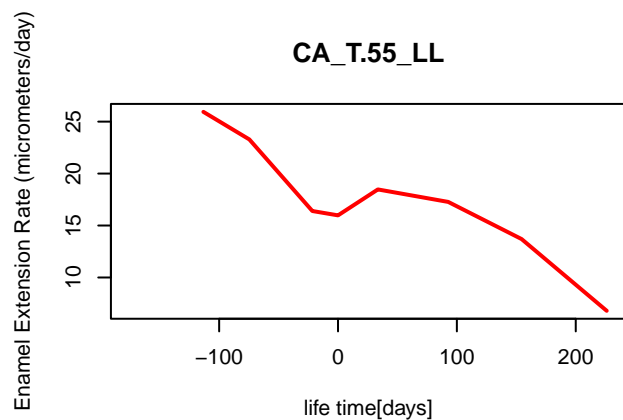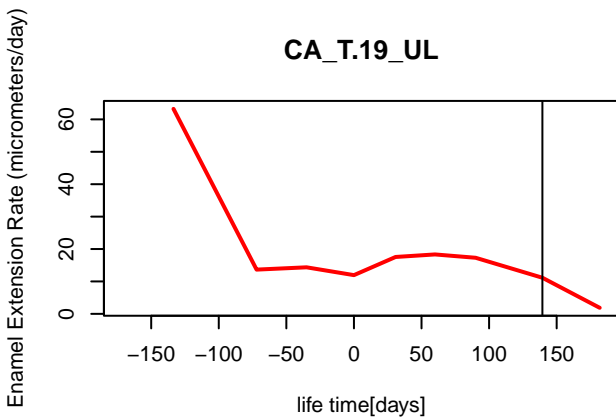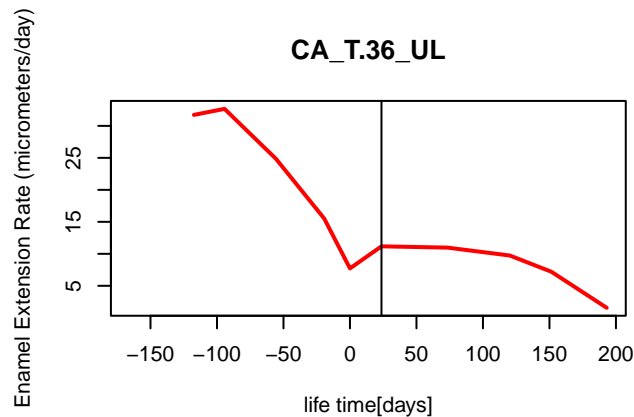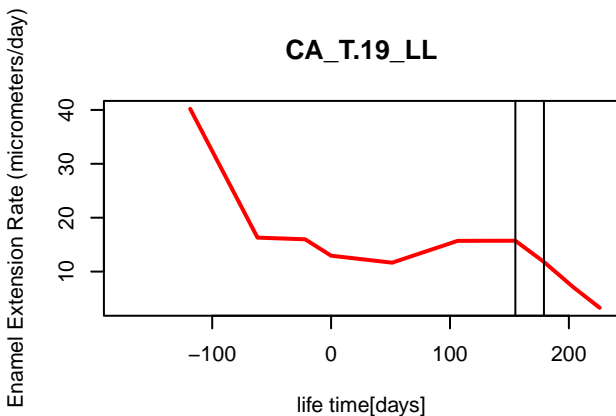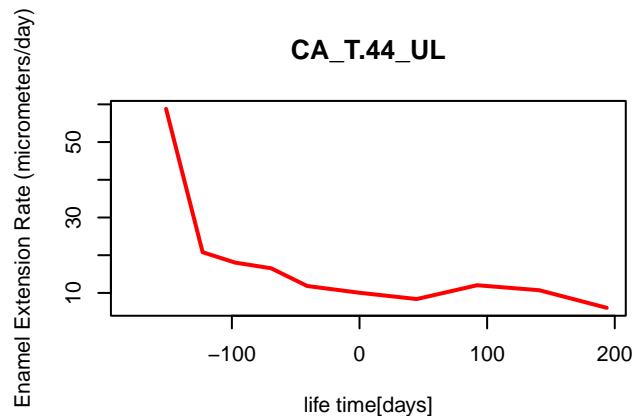

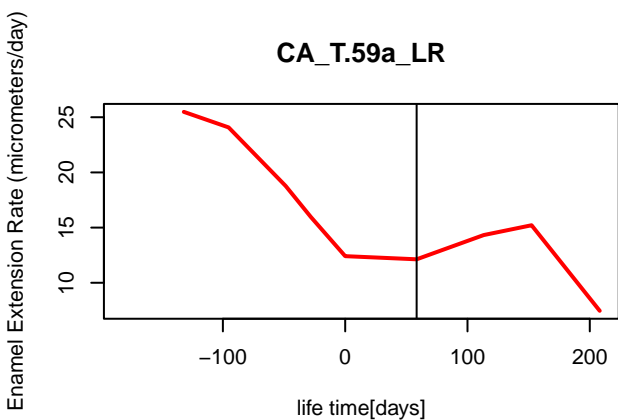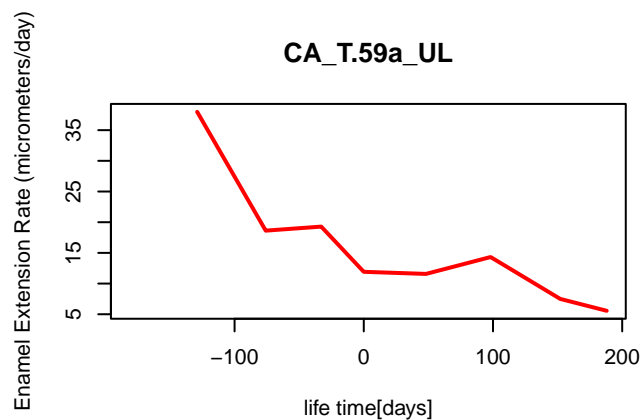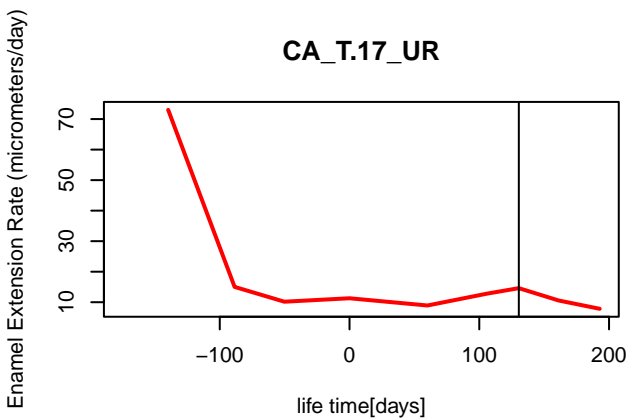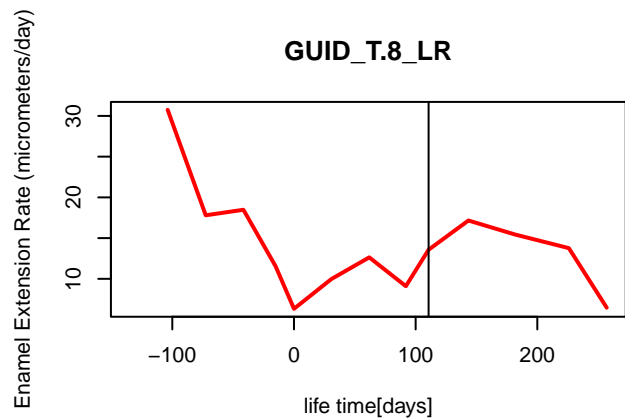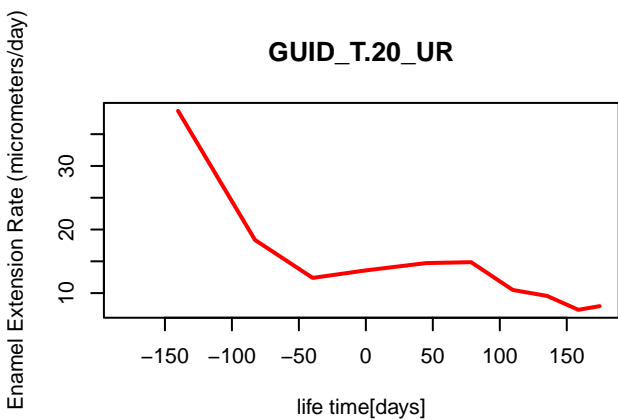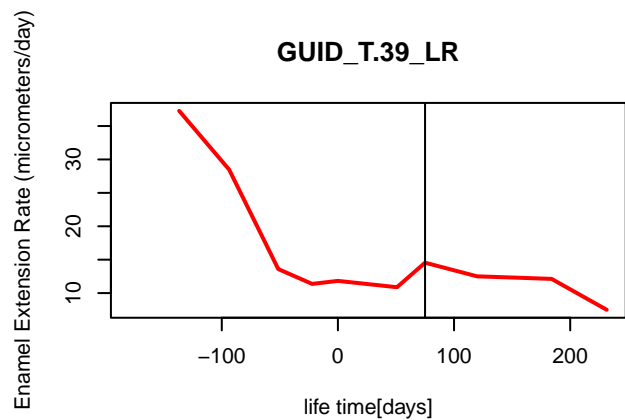

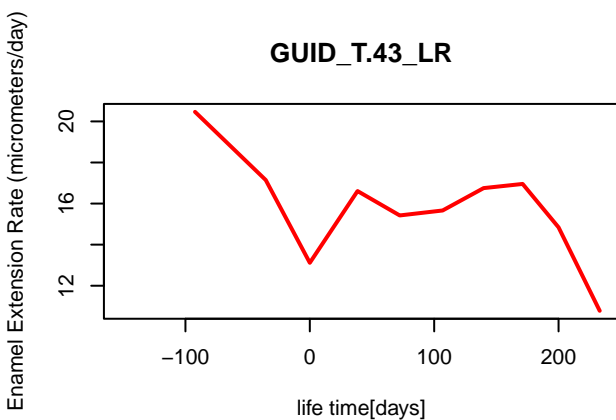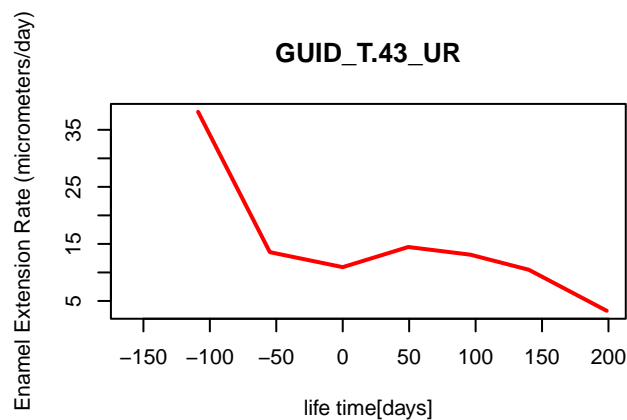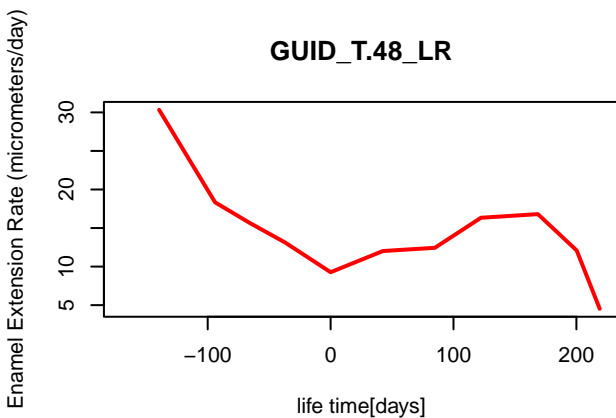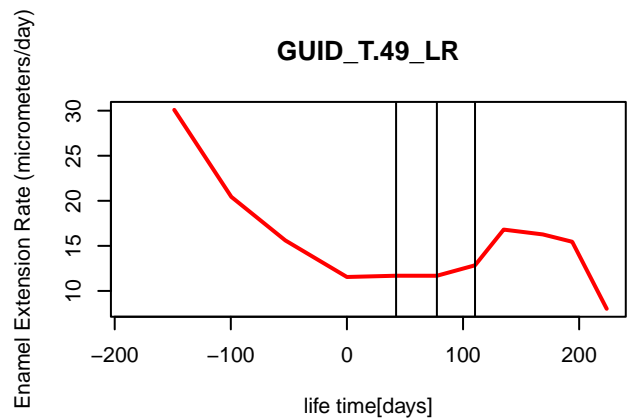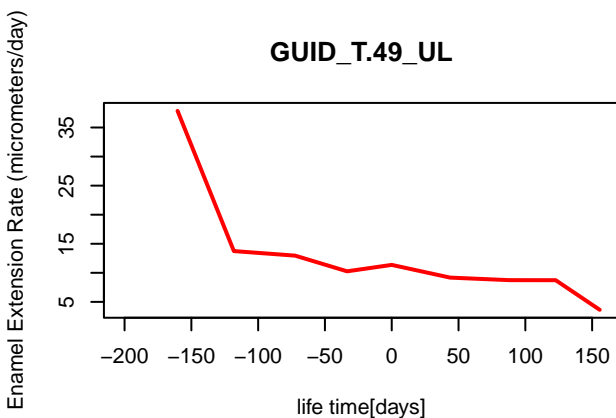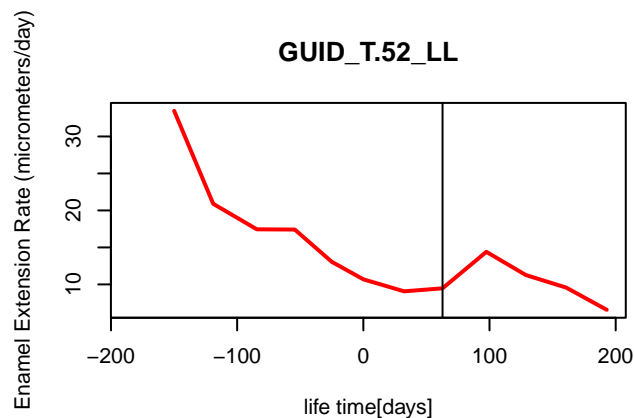

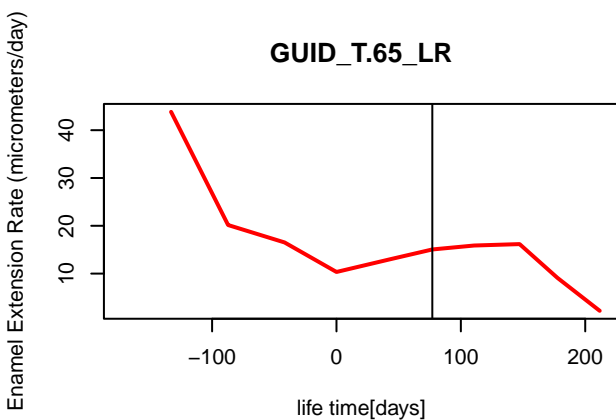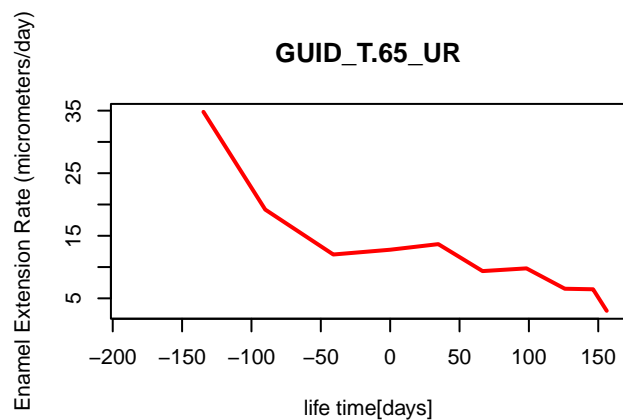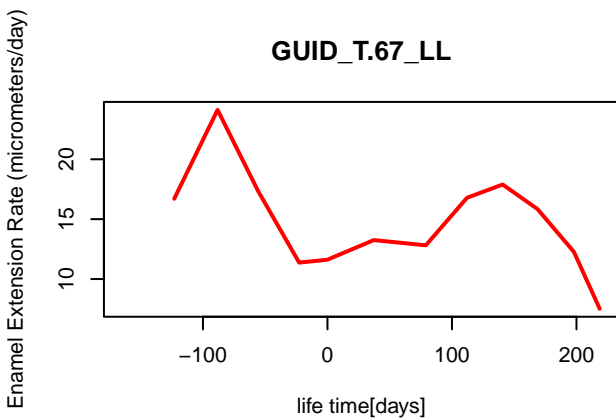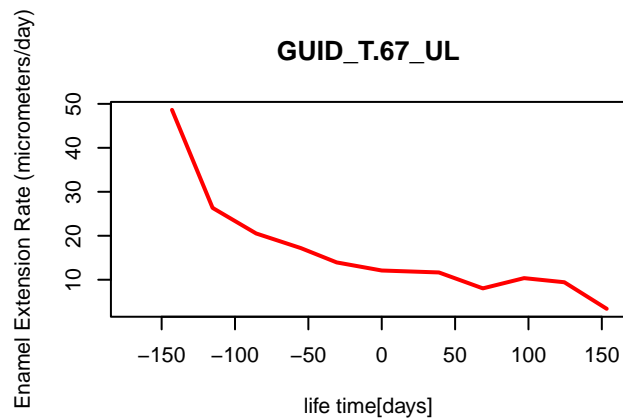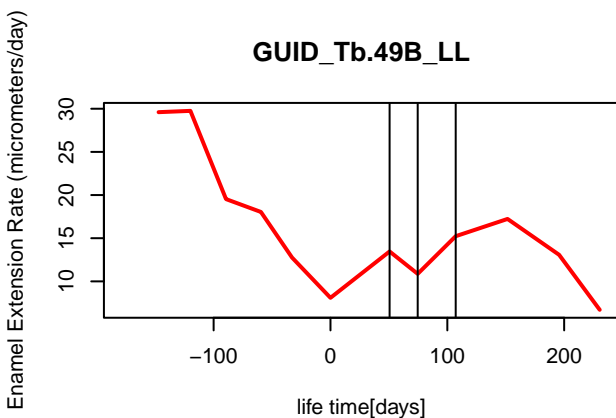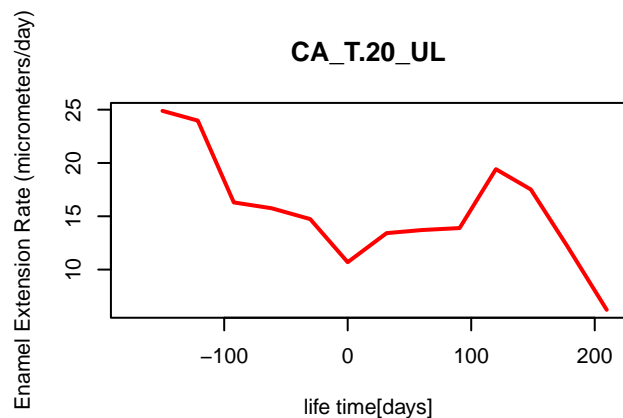

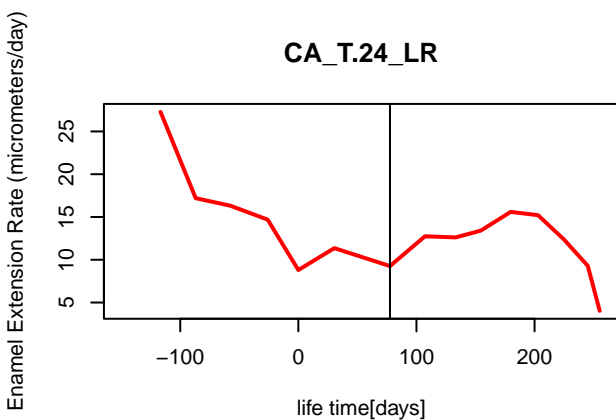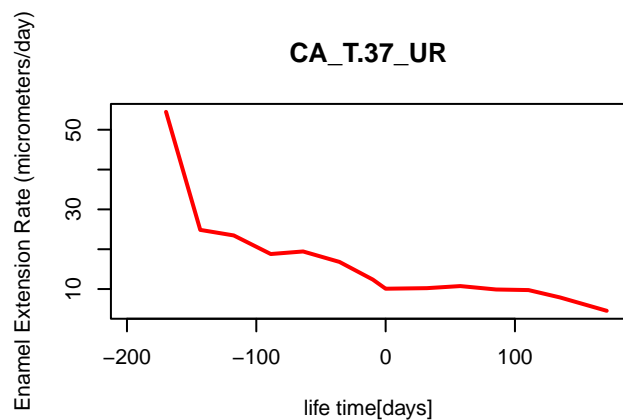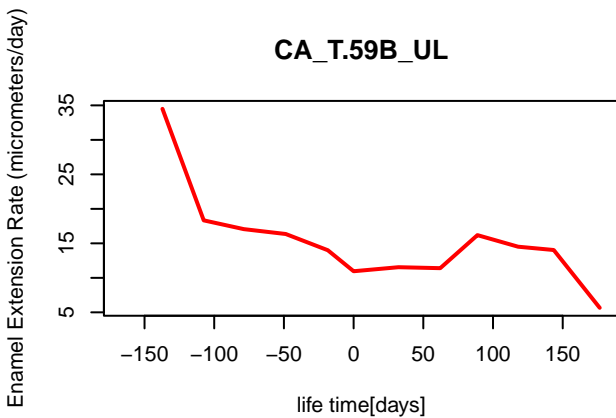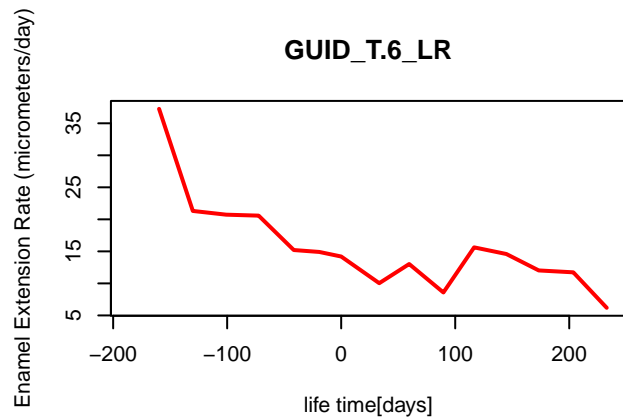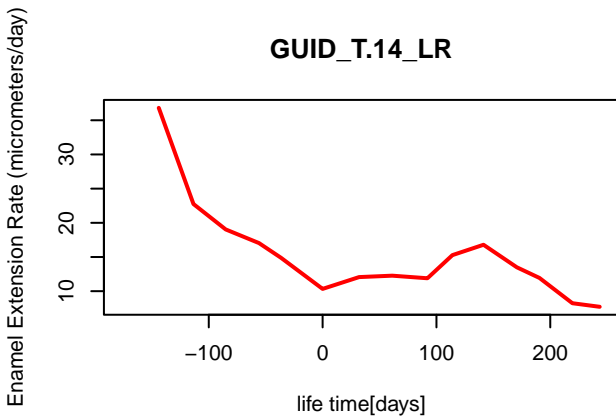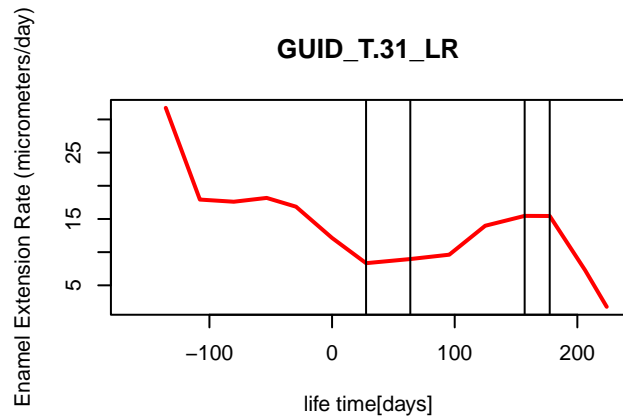

**GUID\_T.33\_UR**

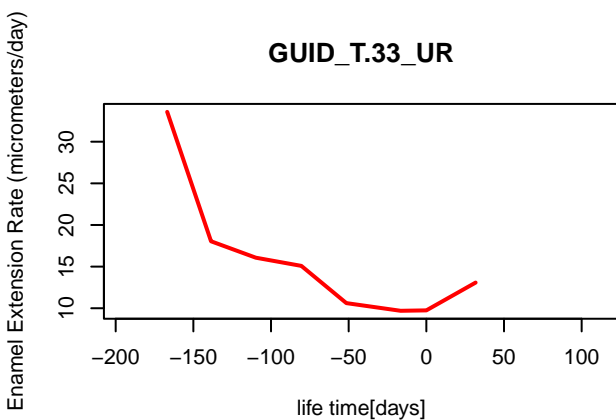

**GUID\_T.54\_UL**

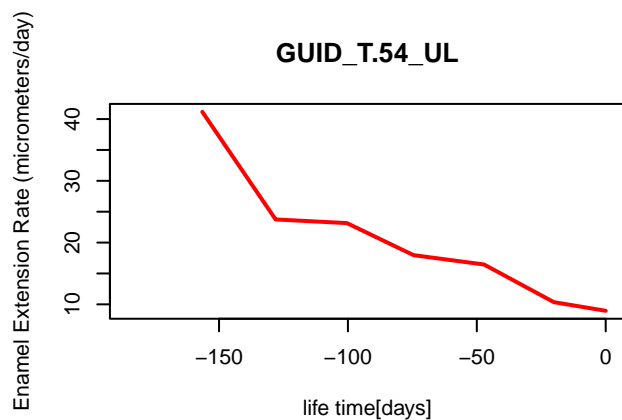

**GUID\_T.61\_LR**

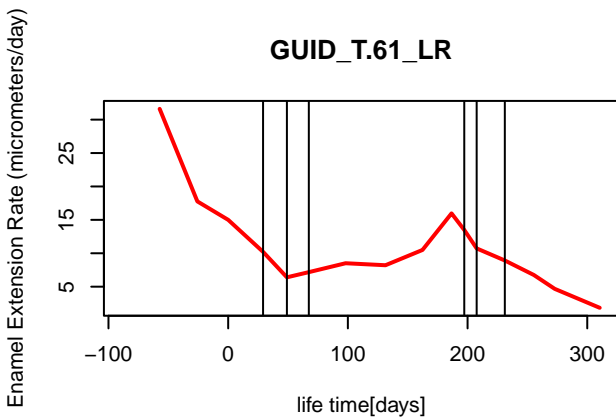

**GUID\_T.89\_UL**

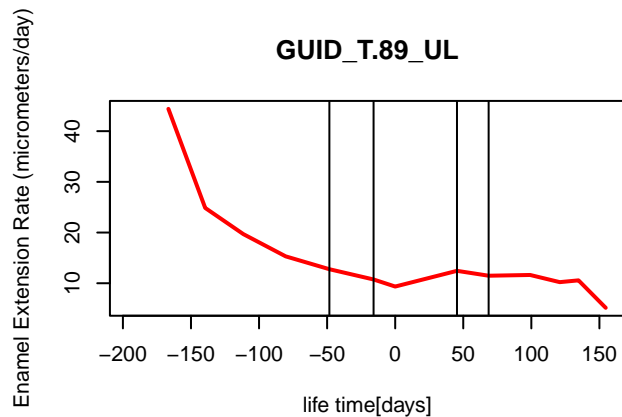

Supplement: S4 Fig — The plots show changes in EER during the lifetime of each individual. Black lines represent the moment of ALs appearance, which are associated with stress events. (PDF) [file pone.0304051.s004.pdf]
